# Vitiligo—Thyroid Disease Association: When, in Whom, and Why Should It Be Suspected? A Systematic Review

**DOI:** 10.3390/jpm12122048

**Published:** 2022-12-12

**Authors:** Ana Maria Chivu, Elena Bălășescu, Larisa Diana Pandia, Roxana Ioana Nedelcu, Alice Brînzea, Gabriela Turcu, Mihaela Antohe, Daniela Adriana Ion

**Affiliations:** 1 2nd Pathophysiology Department, Laboratory of Experimental Medicine and Fundamental Research, Carol Davila University of Medicine and Pharmacy, 37 Dionisie Lupu Street, District 2, 020021 Bucharest, Romania; 2 SanacareVital Clinic, 010161 Bucharest, Romania; 3 Astera Diamed Diabetes & Nutrition and Endocrinology Clinic, 010161 Bucharest, Romania; 4 Derma 360 Clinic, 11273 Bucharest, Romania; 5 Institutul Național de Boli Infecțioase (INBI) Matei Bals, 021105 Bucharest, Romania; 6 Department of Dermatovenerology, Colentina Clinical Hospital, 020125 Bucharest, Romania

**Keywords:** vitiligo, thyroid pathology, autoimmune pathologies, pathophysiology

## Abstract

In most dermatological pathologies, the phenomena observed on the skin are a reflection of internal disorders. In patients with associated acral involvement on the dorsal sides of the hands, this “vitiligo phenotype” may lead to the investigation of certain associated pathologies that sometimes have no obvious clinical impact. To assess the link between skin depigmentation and autoimmune pathologies, we conducted a systematic review involving article selection from the PubMed database. Patients with coexisting thyroid pathologies were found to have a predisposition for developing acral vitiligo and depigmentation of the wrists, and autoimmune thyroid pathologies appeared to be the only coexisting autoimmune or inflammatory diseases in vitiligo patients to show a pattern of distribution. The association of concomitant thyroid dysfunction with depigmentation of the hands was found to be so strong that the absence of depigmented macules on the hands may exclude the coexistence of an autoimmune thyroid pathology. Although the frequency of acral involvement in patients with vitiligo and autoimmune pathologies is higher, the mechanism by which thyroid dysfunction influences this distribution pattern remains incompletely elucidated and requires future studies.

## 1. Introduction

Vitiligo is a dermatological condition that can affect the skin, mucous membranes, and hair, resulting in melanocytic abnormalities [1] that lead to depigmentation [1,2,3]. The pathophysiological mechanisms of this disease are not yet fully elucidated, leading to a lack of specific treatment, which often has repercussions on patients with this type of pathology [3]. Additionally, the emotional impact on the affected population can be high [4,5]. Vitiligo can appear at any age and among all ethnicities, but in most cases, the onset of the disease occurs at a young age (less than 30 years old) [6]. As etiological factors, the involvement of the immune system, as well as the nervous and endocrine systems in a certain genetic context, following exposure to specific environmental factors, has been described in the occurrence of this condition [2,7,8]. Vitiligo can be associated with autoimmune diseases, but not in all cases of depigmentation will such autoimmune pathologies appear [9]. Thyroid damage has also frequently been described in association with vitiligo [2,10]. In this study, we highlight some factors related to this association of thyroid disorders in people with skin depigmentation by conducting a systematic review. The importance of this link between vitiligo and thyroid disorders may lead to targeted personalized therapies able to reduce not only the progression of skin lesions but also the risk of their occurrence.

## 2. Materials and Methods

Considering the association between vitiligo and autoimmune thyroid pathologies, which can evolve into hypothyroidism, hyperthyroidism, or euthyroidism, we conducted a systematic review according to the PRISMA (Preferred Reporting Items for Systematic Review and Meta-analyses) criteria [11] with the aim of studying the association between vitiligo and dysthyroidism from an autoimmune perspective. To this end, we highlight the peculiarities of thyroid dysfunction in patients with vitiligo. We performed article selection from the PubMed database, analyzing articles that appeared between 2012 and 2022.

### 2.1. Search Strategies

The key words initially used were “vitiligo” and “autoimmune thyroidit is”, “anti-TPO”, “hypothyroidism”, “hyperthyroidism”, “dysthyroidism”, “subclinical hypothyroidism”, “subclinical hyperthyroidism”, “Hashimoto disease”, “Graves disease”, “autoimmune polyendocrine syndrome type 3”, and “thyroid autoimmune disorders”. Out of an initial 469 articles, duplicates, articles published more than 10 years ago, articles that did not have the full text freely available, and studies on species other than humans were excluded. Systematic reviews, reviews, meta-analyses, and case report studies were also not analyzed. Inclusion criteria were publications in English, Romanian, French, or Italian; publication dates within the last 10 years; the inclusion of human subjects; and, in terms of study design, case-control studies, cohort studies, multicenter randomized trials, and observational studies (Table 1).

**Figure 1:**
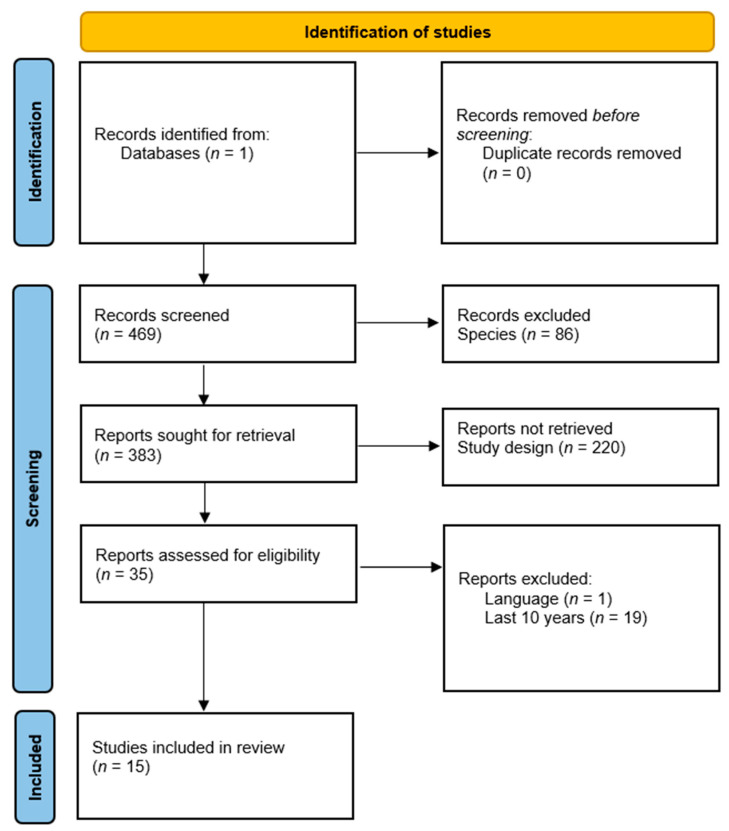
Flow diagram on inclusion criteria.

We found a total of 469 articles published from 1972 to 2021 studying the association between vitiligo and thyroid pathologies. After applying the study design and inclusion criteria with humans as the species of the study subjects as a filter, 35 studies were obtained. After applying the language criteria, 34 articles were obtained. After selecting the last 10 years as the reference period, 17 studies were obtained. One of the studies was excluded because vitiligo was not the subject of the study, which analyzed autoimmunity phenomena but mentioned vitiligo only in possible association with atrophic gastritis and autoimmune thyroid pathologies, highlighting the association of the latter two pathologies. Another study was excluded as it was not freely available. Ultimately, 15 studies were analyzed (Figure 1).

### 2.2. Laboratory Tests Used in Reviewed Studies

Most studies dynamically followed the evolution of clinical–biological parameters aiming to mirror thyroid function and show, in parallel, the anti-melanocyte antibody titers. Anti-TPO (anti-thyroid peroxidase antibodies), anti-tyrosinase, anti-thyroglobulin antibodies, serum TSH (thyroid-stimulating hormone), and fT4 (thyroxine) values were used as parameters to analyze thyroid function. From the perspective of cutaneous autoimmunity, both anti-melanocyte and anti-keratinocyte antibody titers were explored. In one study, the anti-ANA (anti-nuclear) antibody titer was also analyzed [7]. One study focused on measuring the CXCL10 (Th1 immune response prototype chemokine) and CCL (Th2 immune response prototype chemokine) titers as a bridge between autoimmune thyroid pathologies and vitiligo [2]. Most studies have also looked at the hereditary-collateral aspects of autoimmune pathologies, such as familial aggregation, and one study targeted the impact of the genetic component by analyzing 110,814 twins, both monozygotic and dizygotic, among whom co-aggregation and heritability were studied with respect to Hashimoto’s thyroiditis, celiac disease, atrophic gastritis, Graves’ disease, vitiligo, type 1 diabetes mellitus, and Addison’s disease [9]. In addition, from a genetic point of view, the relationship between MIF (macrophage inhibitory factor) gene polymorphism and the prognosis of Graves’ disease in the progression to a severe form was studied in the context of an association with vitiligo [10].

### 2.3. Interventions

Some studies have examined the development of multiple autoimmune reactions following interventions. These interventions included anti-tumor therapies such as the use of nivolumab in the treatment of melanoma [12]. Nivolumab is an anti-PD-1 (anti-programmed death-1) monoclonal antibody; part of the checkpoint inhibitor family; and used in anti-tumor therapy for advanced kidney cancer, melanoma, and lung cancer [12]. Another example of an intervention is combination treatment using checkpoint inhibitors and IL-2 (interleukin-2) in the treatment of metastatic melanoma and metastatic renal cell carcinoma [13]. These autoimmune reactions occurring in the context of anti-tumor therapies have been linked to favorable tumor prognosis (Figure S1).

### 2.4. Data Extraction

The studies included in this review were analyzed on the basis of inclusion and exclusion criteria, and data were extracted by reading the title, abstract, and full text. Data extracted from each study included the name of the first author, the full title of the article, the year of publication of the article, the type of study, the characteristics of the sample (the number of patients included in the study, with the subsequent specification of the particularities of their selection, including the number of subjects, number of groups, and the criteria of group formation), the parameters followed in the study, and the results obtained (Tables S1 and S2).

## 3. Results

A total of 2462 vitiligo patients with different phenotypes, from different ethnic groups, and with different disease courses were included in the analysis of all studies. A total of 110,814 monozygotic and dizygotic twins were included in the studies, and the association of autoimmune pathologies among them was analyzed. The occurrence, evolution, and association with vitiligo in particular groups of patients were also analyzed. The sample included 1535 cancer patients, including 623 with metastatic melanoma, 919 patients with metastatic renal cell carcinoma, 7 patients with an association of these two cancers in metastatic form, 290 patients with alopecia areata, 100 patients with lichen sclerosus, and 481 patients with Graves’ disease.

Vitiligo was analyzed both in terms of its influence on responses to anti-tumor therapy and as an influencing factor on thyroid autoimmune pathology. The onset of vitiligo appears to be associated with certain patterns of skin distribution and a specific temporal evolution. These studies once again highlighted the multifactorial genesis of vitiligo involving both genetic polymorphisms [14,15,16] and lifetime exposure to environmental factors [17].

A higher prevalence of autoimmune thyroid pathologies was observed among patients with vitiligo compared to the general population [18,19,20,21,22]. A single-center retrospective observational study highlighted that of all the autoimmune pathologies associated with vitiligo, the most common is autoimmune thyroiditis [23]. In addition to the simple association of these pathologies, the influence of thyroid dysfunction on vitiligo progression is worth exploring [24,25,26]. In a case-control study, the titers of anti-thyroid antibodies in stable and progressive vitiligo were monitored [18]. Progressive vitiligo was considered a form of the disease in which new lesions had appeared or pre-existing lesions had spread in the last three months; in another study, subclinical hypothyroidism was detected in 27.8% of patients and high levels of anti-TPO antibodies in 40.3% of patients [23]. A cohort study including 434 patients aimed to investigate the prevalence of thyroid dysfunction in correlation with anti-TPO antibody titers in patients with NSV (nonsegmental vitiligo) in order to investigate the usefulness of screening tests for thyroid pathologies in patients with vitiligo [27]. Anti-TPO antibodies are considered a sensitive way to detect early subclinical forms of autoimmune thyroiditis and, at the same time, identify patients at increased risk of developing autoimmune thyroid pathologies [28]. Out of 434 patients, 43 were already diagnosed with thyroid dysfunction, 27 were previously screened for thyroid pathologies with a negative result in the last three months, and the remaining patients enrolled in the study were screened for thyroid pathologies. TSH, fT4, and anti-TPO levels were analyzed in all 364 patients who did not already have a detected thyroid dysfunction or did not have a screening performed in the last three months [27]. The diagnosis of subclinical hypothyroidism involves elevated TSH levels and a normal serum fT4 level, while overt clinical hypothyroidism involves elevated TSH and a low fT4 level. On the other hand, subclinical hyperthyroidism consists of a low TSH level associated with normal fT4 values, and clinically manifested hyperthyroidism involves a low TSH associated with an increased fT4 level. The conclusion of this study was that thyroid dysfunction is more common among patients with NSV than among the general population [27]. However, it should be noted that the number of cases of newly diagnosed hyperthyroidism along with subclinical forms in the study population was relatively small, and most of the thyroid dysfunctions were already discovered by a physician to whom the patient had already presented with symptoms [27]. Thus, screening for thyroid pathologies is recommended in selected population groups, specifically, those with a positive family history of thyroid dysfunction and older women, as thyroid pathologies are prevalent among these two groups [27]. In agreement with this study, N. Van Geel et al. noted that among vitiligo patients, women are more frequently concomitantly affected by other autoimmune or inflammatory pathologies compared to men, noting that this difference was the most evident in the case of autoimmune thyroiditis [23]. To assess the prevalence of clinically overt hypothyroidism between these 434 vitiligo patients, the results were compared with the prevalence in the general population based on the Wickham prospective study analyzing the incidence of hypothyroidism through a 20-year follow-up [29]. The risk of developing clinically overt hypothyroidism was correlated with anti-TPO antibody titers as follows. At 20-year follow up, the prevalence was 23% if anti-TPO titers were weakly positive (100–400 kU/L), 33% if titers were moderately positive (400–800 kU/L), and 53% if titers were strongly positive [29].

In another study, associations between vitiligo and anti-TPO (25.6%), anti-thyroglobulin (23.4%), antinuclear (16.8%), and anti-parietal cell (7.8%) antibodies were identified, noting that a total of 74 of the patients had autoimmune comorbidities (41.5%), mainly autoimmune thyroiditis [28]. The presence of anti-TPO antibodies was significantly associated with a long duration of vitiligo [30,31] and a positive family history of vitiligo, and anti-thyroglobulin antibodies were significantly associated with the female gender [28,31].

## 4. Discussion

A higher prevalence of autoimmune thyroid pathologies was observed among patients with vitiligo compared to the general population [32,33]; such pathologies most commonly present as thyrotoxicosis (Graves’ disease and hashitoxicosis) and hypothyroidism [18]. Given the strong association between anti-TPO titers and the risk of developing clinically overt hypothyroidism, screening is recommended in patients with intensely positive antibody titers and NSV, and annual thyroid function testing is warranted in patients with antibody titers > 800 kU/L at initial screening [27].

One of the most important considerations when recommending screening for thyroid dysfunction in patients with vitiligo is that screening asymptomatic individuals and treating the underlying pathology early can lead to much better outcomes in terms of prognosis and progression of the skin pathology compared to subjects who were not screened and presented to the physician with already clinically manifested skin pathologies [27].

Although some large population-based studies showed a low association between vitiligo and thyroid pathologies [29], the availability of early therapeutic interventions and subsequently good outcomes among patients supports the screening of the vitiligo population for thyroid dysfunction [34,35].

### 4.1. Temporal Association

Usually, vitiligo precedes the onset of thyroid dysfunction [18,31,36,37]. However, in a cohort study of 434 patients with NSV, a positive family history of vitiligo was observed in 102 patients, and a history of thyroid dysfunction was observed in 43 of them, 26 of whom experienced the onset of a thyroid pathology before the onset of vitiligo. However, 13 patients experienced vitiligo onset before the onset of a thyroid pathology; in the remaining four patients, the temporal association of the two pathologies in terms of onset was not specified [27].

### 4.2. Characteristics of Age Groups

It was shown that thyroid dysfunction with an autoimmune etiology is more common in patients over 30 years of age [18,36]. An association was also found between thyroid dysfunction and a positive family history of thyroid pathology, as well as with older women [27]. The presence of circulating antibodies was correlated with both a long duration of vitiligo and the onset of vitiligo at an older age, but antibody titers were not correlated with the progression, regression, or stability of vitiligo. A retrospective observational study of 700 vitiligo patients highlighted the lack of a clear difference in response to therapy or vitiligo activity in patients with other concomitant autoimmune or autoinflammatory pathologies [23] and found that forms of NSV are more commonly associated with a personal or family history of autoimmunity compared to forms of SV (segmental vitiligo) [23,38].

It was observed that, in the case of a pediatric onset of vitiligo, the clinical form of the presentation included several areas of depigmentation, with SV being predominant [39]; patients with the pediatric onset of vitiligo were more frequently associated with allergic pathologies and, less frequently, thyroid pathologies than those with adult-onset vitiligo [40,41]. Gender, phototype, age of vitiligo onset, duration of disease, presence of a stressful event related to depigmentation, disease progression or stationary form, family history of vitiligo, body surface area affected, and family history of thyroid pathologies were explored as variables and associated with the presence or absence of thyroid pathology in each patient. Thus, in the pediatric-onset group, analysis of these variables revealed that two variables were significantly associated with the concomitant presence of thyroid pathology: a long duration of vitiligo and a family history of thyroid pathologies [39]. In the group with adult onset of vitiligo, the only variable that was significantly and positively correlated with the presence of thyroid pathologies was the sex of the patient [39].

A prospective observational study was performed on 679 patients, aiming to compare the clinical features of patients with prepubertal and post-pubertal onset of NSV [6]. Forms of vitiligo universalis were observed only in post-pubertal-onset NSV and thyroid pathologies, and circulating anti-thyroid antibodies were more common in patients with a post-pubertal onset of NSV [6]. A history of thyroid pathologies and acrofacial type were more commonly associated with a post-pubertal onset [6]. In terms of lesional distribution, the trunk and limbs were the most frequently spared in patients with post-pubertal onset, and, in contrast, the head and neck were the most commonly affected sites in post-pubertal onset vitiligo [6]. Stressful events and stress as a factor related to the onset, long duration of the disease, acro-facial subtype, and the presence of autoimmune thyroiditis or anti-thyroid antibodies were associated with post-pubertal onset. On the other hand, it was observed that patients with a pre-pubertal onset of vitiligo were more frequently associated with episodes of spontaneous repigmentation, generalized vitiligo subtype, the presence of halo nevi, a family history of premature hair graying, a family history of vitiligo, the concomitant presence of atopic dermatitis, and a family history of other autoimmune pathologies [6]. Pruritus was more commonly described among the post-pubertal onset group than among the pre-pubertal onset group [6].

R. Speeckaert et al. found that in patients with vitiligo who had an earlier onset, the lower limbs were more frequently affected, whereas onset in old age was associated with damage to the upper extremities [42]. A possible explanation for this association may be that younger patients are more prone to both microtrauma and macrotrauma of the lower extremities due to outdoor sports activities. In contrast, in older age, pressure from the elbows in the context of office work or home activities tends to have a greater impact on the upper extremities, with more frequent trauma to the hands and wrists. Depigmentation in the mandibular and chin areas were more common in men than women, and, in contrast, underarm depigmentation was twice as common in women, a distribution that can be explained by the traumatic stimulus of razor blade use in those areas, as both injuries were associated with adulthood. The reason for the association of perioral involvement with an older age of onset of vitiligo and periocular involvement with an earlier age remains unclear. One theory that could, however, explain this distribution is the association of atopic eczema and allergic rhinitis with younger patients, who tend to frequently touch their periorbital areas through rubbing phenomena [42].

It has been observed that patients with a pre-pubertal onset of vitiligo have a higher chance of spontaneous repigmentation. This factor calls into question the choice of early therapeutic intervention in the course of the disease, while taking into account that therapeutic interventions are more effective the closer they are initiated to the onset of the pathology and are less effective on old lesions [6].

### 4.3. Particular Features of the Body Distribution of Vitiligo according to Autoimmune Thyroid Pathology

**Figure 2:**
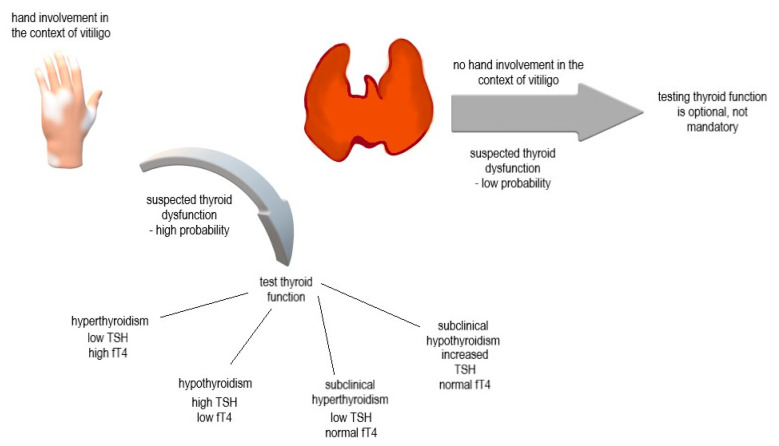
Hand depigmentation, as a clinical landmark in vitiligo, is highly suggestive of associated thyroid dysfunction.

A retrospective observational study of 700 vitiligo patients reported that the percentage of total body surface area affected by the disease is significantly higher in the presence of thyroid pathology, which, in turn, is more common among women [23]. The overall concomitant presence of other autoimmune or inflammatory pathologies does not seem to significantly influence depigmentation in vitiligo, with the exception of autoimmune thyroid pathologies (and psoriasis), which significantly influence the percentage of skin depigmentation in vitiligo. In terms of gender, the total body surface area involvement in vitiligo is greater in women with an associated autoimmune pathology than in women without an associated autoimmune thyroid pathology. However, in men, the association of thyroid pathology does not appear to significantly influence the course of vitiligo [23]. Additionally, patients with coexisting thyroid pathologies showed a particular predisposition to develop acral vitiligo and depigmentation of the joints [23]. Moreover, the presence of an associated autoimmune or inflammatory pathology seems to influence the clinical profiles of vitiligo patients [43], and it is argued that in the presence of an autoimmune thyroid pathology, the form of vitiligo tends to be more extensive, with a particular predilection towards areas subject to microtrauma. Patients with vitiligo and other concomitant autoimmune or inflammatory pathologies in the presence of a positive family history of autoimmune or inflammatory pathologies had the largest skin area affected by depigmentation in vitiligo. The most common distribution of skin involvement in vitiligo patients with associated autoimmune or inflammatory pathologies was the face, followed by the acral areas [23,43]. The prevalence of acral involvement among patients with vitiligo is higher in those with an associated autoimmune thyroid pathology than in those without a concomitant thyroid pathology. Comparatively, hands and wrists were more frequently affected by depigmentation among patients with associated autoimmune or inflammatory diseases compared to those without these associated pathologies; this type of damage is consistent with the Koebner phenomenon caused by microtrauma from friction. The coexistence of autoimmune thyroid pathology with vitiligo is so strongly associated with acral depigmentation, especially in the hands, that the absence of depigmentation in these areas may be a criterion for excluding the presence of a thyroid pathology [23] (Figure 2).

Differences in the distribution of skin depigmentation were statistically significant in patients with associated autoimmune thyroid pathologies, with a higher frequency of hand depigmentation observed in both women and men early after vitiligo onset [23]. Knees, elbows, and hips were less frequently affected in patients with vitiligo and other associated autoimmune or inflammatory pathologies than in patients with vitiligo alone [23]. Another aspect related to the distribution pattern of depigmentation in patients with vitiligo is that concomitant depigmentation of both wrists and ankles was associated with the coexistence of other autoimmune pathologies [42]. It was also found that depigmentation of the legs without that of the hands was mainly observed among patients lacking associated autoimmune pathologies [42].

Even if the predilection of acral involvement (i.e., areas more prone to the Koebner phenomenon) could be explained in patients with associated autoimmune pathologies by the loss of peripheral tolerance and the consequent increase of the inflammatory response, the reason why only thyroid pathologies seem to be associated with a specific distribution pattern remains incompletely elucidated [23]. It is well known that both minor pressure or friction trauma and major trauma leading to linear or punctiform lesions can cause depigmentation of the lesions via the Koebner phenomenon [42,44]. These non-specific stimuli most likely initiate an immune response that leads, in vitiligo patients, to the recruitment of specific CD8 lymphocytes directed against melanocytes [42,45,46].

Another study of 79 patients evaluated the association between the clinical distribution pattern of depigmented macules and the level of serum antibodies, including anti-melanocyte, anti-thyroid, and anti-keratinocyte antibodies [47]. Antibodies against tyrosinase, tyrosine hydroxylase, thyroperoxidase, thyroglobulin, and keratinocytes were detected with a frequency of 11%, 22%, 18%, 24%, and 27%, respectively. The presence of antibodies was more frequently associated with forms of NSV (50–67%) than with forms of SV (0–17%). Additionally, antibodies were predominantly detected in patients with a shorter time to disease onset [47]. Anti-thyroglobulin and anti-TPO antibodies were observed in 23/79 patients (29%), but only one patient presented clinically overt autoimmune hypothyroidism. The presence of anti-thyroid antibodies indicates the potential development of clinically overt thyroid dysfunction and corresponds to a recommendation for follow-up of TSH and fT4 levels over time. Notably, a study conducted on the Indian population identified an association of only 3% between other autoimmune pathologies and vitiligo, which conflicts with other Western European population studies that detected a frequency of 30%. This result could highlight the potentially different genetic susceptibility levels between populations or the absence of the triggering role of environmental factors [47]. Furthermore, another retrospective study of 2441 vitiligo patients conducted in the United States reported differences in the rate of coexisting autoimmune pathologies according to race, identifying a higher prevalence among Caucasians (38.4%) than among African Americans, Hispanics, or Asians [28].

From a genetic perspective, large-scale genome-wide association studies have identified in generalized vitiligo an association with a number of loci encoding regulatory immunoproteins that do not belong to the major histocompatibility complex [28]. The different association rates of autoimmune comorbidities and circulating antibodies detected in various epidemiological studies could be explained by variations in gene expression between different populations or by the action of different environmental factors as triggers [17,28,48]. Based on information from the Swedish twin registry, the co-aggregation of autoimmune pathologies was found to be more frequent among monozygotic twins [9]. Another argument for the importance of the genetic component was made by K. Ezzedine et al., who reported a strong association between the pre-pubertal onset of vitiligo and a family history of autoimmune pathologies, highlighting that a pre-pubertal onset of vitiligo implies a stronger genetic component than a post-pubertal onset [6]. Furthermore, the hypothesis that the age of onset of vitiligo is influenced by the genetic component is supported by the fact that a family and personal history of atopic dermatitis are more prevalent among patients with pre-pubertal onset [6].

Recently, the importance of the Th1-type immune response in the pathogenesis of vitiligo, as well as the C-X-C Motif Receptor 3 (CXCR3) and its corresponding chemokine, CXCL10, was highlighted. Both were proposed to represent new potential therapeutic targets [2]. CXCL10 is a gamma-interferon-induced chemokine in neutrophils, lymphocytes, endothelial cells, and thyrocytes [2]. Moreover, high serum and tissue levels of CXCL10 have been observed in organ-specific autoimmune pathologies such as type 1 diabetes mellitus, Graves’ disease, or other autoimmune thyroiditis, and Graves’ ophthalmopathy, as well as in systemic autoimmune pathologies such as rheumatoid arthritis, systemic lupus erythematosus, systemic sclerosis, and cryoglobulinaemia in the context of hepatitis C [2]. Common elements of major importance in the pathogenesis of NSV and thyroid autoimmune pathologies are serum CXCL10 and CCL2 ligand values, which were measured in 50 patients with NSV, 40 with NSV and thyroid autoimmune pathology, 50 sex- and age-matched patients with autoimmune thyroiditis (control group 1), and 40 sex- and age-matched patients with autoimmune thyroiditis (AT) and without NSV (control group 2). Serum CXCL10 levels were significantly higher in control group 2 compared to control group 1, patients with NSV had a higher serum CXCL10 level than those in control group 1 or 2, and patients with NSV and AT had higher CXCL10 levels than those in both the control groups [2]. These results are important in highlighting the importance of the Th1-type response in the pathogenesis of these autoimmune pathologies, with the highest CXCL10 levels observed in patients with VNS who also associated BP with hypothyroidism. However, further studies are needed to determine whether CXCL10 can be pursued as a clinical marker of NSV [2].

Taken as a whole, thyroid pathologies appear to be the only autoimmune or inflammatory diseases associated with vitiligo that show a particular pattern of distribution.

### 4.4. Prognosis of the Association between Vitiligo, Autoimmune Thyroid Pathologies, and Oncological Treatment

Over the past 10 years, cell checkpoint inhibitor therapy has led to major therapeutic breakthroughs in cancer pathologies. In patients receiving this type of treatment, autoimmune reactions occur as a side effect and are known as immune-related adverse events (irAEs), with corresponding rash, vitiligo, immune pneumopathies, hepatitis, thyroiditis, nephritis, colitis, etc. Skin depigmentation was observed in patients with melanoma treated using interferon, and the development of vitiligo was found to correlate with prolonged disease control [49,50]. A retrospective observational cohort study examining the survival of patients with melanoma and stage 4 renal cell carcinoma linked the occurrence of irAEs after checkpoint inhibitor treatment and IL-2 as a positive prognostic factor for therapeutic response [13]. Tumor control was 71% in patients who combined irAEs. However, in those who did not develop irAEs, following therapy, tumor control was 56% [13]. Tumor control was assessed by correlating the complete response, partial response, and pathology stability over time. The survival rate was statistically higher in the context of developing irAEs during or after IL-2 therapy, with a mean of 48 months in patients with metastatic melanoma versus 18 months in those who did not develop irAEs. In patients with metastatic renal cell carcinoma, the median survival was 60 months for those who developed irAEs versus 40 months for patients who did not develop irAEs. The most common irAE to occur following IL-2 therapy was vitiligo (70% of irAEs were associated with IL-2 therapy). Other autoimmune reactions reported following calcineurin inhibitor therapy include psoriasis, colitis, thyroiditis, hepatitis, and hemolytic anemia. The therapeutic response was 34.5% in patients who developed irAEs and 21.8% in patients who did not develop irAEs [13].

In melanoma patients, vitiligo-like phenomena may occur [51,52] spontaneously because both normal and uncontrolled proliferating melanocytes share certain common surface markers [53], such as glycoprotein 100, which causes the immune system to react against both malignant cells, in an attempt to limit local invasion phenomena, and secondarily against normal melanocytes, whose apoptosis [54,55] is clinically reflected by the appearance of depigmented macules even at a distance from the melanoma lesions [51,53,56]. This reaction is a positive prognostic factor in cancer patients as it reveals activation, integrity, and active attack of the immune system [50].

An autoimmune pathology associated with vitiligo and autoimmune thyroiditis is alopecia areata [57,58], and another is lichen sclerosus [7,59].

In a prospective interventional study, the clinical response to photodynamic therapy for genital lichen sclerosus and the possible influence of this therapy on antibody titers were observed [7]. The most common autoimmune pathology associated with lichen sclerosus was thyroiditis, followed by vitiligo and arthritis. No statistically significant difference in anti-thyroid antibody titers was observed after photodynamic therapy, but a statistically significant difference was identified for anti-nuclear antibodies; improvement of symptomatology after therapeutic intervention was also observed [7].

In a case-control study, the influence of Macrophage Migration Inhibitory Factor (MIF) gene polymorphism on the clinical severity of Graves’ disease was investigated [10]. The MIF gene encodes a cytokine of the same name, which is produced in epithelial cells and participates in both innate and adaptive immune responses. The presence of vitiligo as an autoimmune-associated pathology was correlated with a moderately severe form of Graves’ disease [10,60], and the C/G and G/C alleles within the MIF gene polymorphism were correlated with the existence of vitiligo in patients with untreated Graves’ disease [10].

## 5. Conclusions

The main conclusions of this review are as follows:

- Autoimmune thyroid disease is more common among people with NSV than among the general population. Women with vitiligo more frequently presented autoimmune pathologies than men, with a higher prevalence of autoimmune thyroiditis.
- Screening for thyroid pathologies is particularly recommended for older patients with a family history of thyroid pathologies. The most common antibodies in vitiligo patients are anti-thyroid antibodies (ATPO, anti-Tg). Screening of asymptomatic patients together with early treatment of the underlying pathology leads to better results in terms of the prognosis of the pathology and the evolution of the patients compared to subjects who were not screened beforehand and presented to the doctor with an already-clinically-manifested pathology.
- Anti-TPO antibodies are a sensitive way to detect subclinical forms of autoimmune thyroiditis early. ATPO titers do not seem to correlate with the extent of vitiligo or a particular subtype of vitiligo. On the other hand, the percentage of total body surface area affected by vitiligo was found to be significantly higher in the presence of thyroid pathology, which, in turn, is more common among women.
- Patients with coexisting thyroid pathologies have a predisposition to developing acral vitiligo and depigmentation of the wrists. The association between concomitant thyroid dysfunction and depigmentation of the hands is so strong that the absence of depigmented macules on the hands may exclude the coexistence of autoimmune thyroid pathology. Taken together, autoimmune thyroid pathologies appear to be the only coexisting autoimmune or inflammatory diseases in vitiligo patients to show a pattern of distribution. Although the frequency of acral involvement in patients with associated autoimmune pathologies is higher, the mechanism by which thyroid pathology influences this distribution pattern remains incompletely elucidated. One possible explanation is that these areas are more prone to the Koebner phenomenon, which, in patients with associated autoimmune pathologies, can occur through a loss of peripheral tolerance and a resulting increase in the inflammatory response. These non-specific stimuli most likely initiate an immune response that causes the recruitment of CD8+ lymphocytes directed against melanocytes.
- Vitiligo tends to precede the onset of thyroid pathology, and patients who lack other associated autoimmune or inflammatory pathologies but have a positive family history of these pathologies tend to have an early onset of vitiligo.
- The chances of repigmentation among patients with vitiligo and associated autoimmune pathologies are not statistically significant compared to the chances among patients without associated autoimmune pathologies.
- Both autoimmune thyroiditis and vitiligo can be considered immune adverse reactions during antitumor therapy, and both are positive prognostic factors for therapeutic response and survival.
- Vitiligo should be considered an integrative pathology with which other autoimmune pathologies may be associated. This framework should form the basis of clinical reasoning and guide relevant screening modalities in order to detect the onset of other diseases as early as possible. This process should be understood as reciprocal so that more attention is given to the skin in order to detect possible depigmented macules in patients with associated autoimmune polyglandular syndromes, pernicious anemia, rheumatological pathologies, alopecia areata, or thyroiditis of an autoimmune etiology.

## Figures and Tables

**Table 1:** Inclusion criteria.

Parameter Used	Inclusion Criteria	Exclusion Criteria
Language	English, French, Romanian, and Italian	Any language not already listed
Access	Free access to the full text	Access to the full text, financially conditional
Species	Human subjects	Any species other than Human
Type of Study	Observational, cohort, case-control, single-center or multicenter randomized studies	Review, meta-analysis, systematic review, case report studies
Publication period	Studies published in the last 10 years (2012–2022)	Studies published before 2012
Duplicate	No	Yes

Selection criteria for articles reviewed.

## Data Availability

Not applicable.

## Supplementary Materials

The following supporting information can be downloaded at: https://www.mdpi.com/article/10.3390/jpm12122048/s1, Figure S1: Side effects of Nivolumab, seen as a positive prognostic factor for the therapeutic response. irAEs- immune related adverse events; Table S1: Characteristics of included studies; Table S2: Results from analyzed studies.
