# Supplementary material for: Vitiligo—Thyroid Disease Association: When, in Whom, and Why Should It Be Suspected? A Systematic Review
Source: J Pers Med. 2022 Dec 12;12(12):2048. doi: 10.3390/jpm12122048 (PMC9785784; doi:10.3390/jpm12122048)
Supplement: Supplementary file 1 [file jpm-12-02048-s001.zip › Figure S1. Side effects of Nivolumab, seen as a positive prognostic factor for the therapeutic response..pdf]

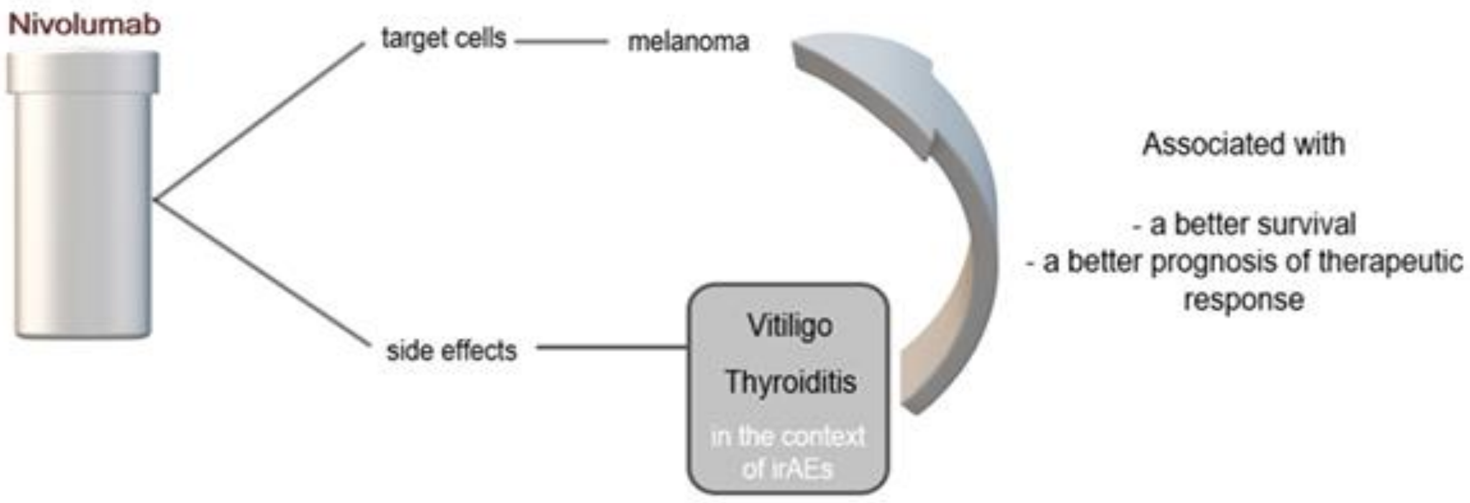


**Figure S1.** Side effects of Nivolumab, seen as a positive prognostic factor for the therapeutic response. irAEs- immune related adverse events.
