# Supplementary material for: Vitiligo—Thyroid Disease Association: When, in Whom, and Why Should It Be Suspected? A Systematic Review
Source: J Pers Med. 2022 Dec 12;12(12):2048. doi: 10.3390/jpm12122048 (PMC9785784; doi:10.3390/jpm12122048)
Supplement: Supplementary file 1 [file jpm-12-02048-s001.zip › Table S1.pdf]

Table S1. Characteristics of included studies.

No.	Authors	Year	Type of study	Number of patients	Tracked Pramets
1	Yazdanpanah, M.J., *et al.*	2016	Case-control	72 patients with vitiligo	ATPO, fT4, TSH
2	Nicolaidou, E., *et al.*	2016	Transversal	233 patients with vitiligo	Family history of vitigo, thyroid pathology, sex phenotype, duration of disease and course, onset, progression, stressful events
3	Freeman-Keller, M., *et al.*	2017	Retrospective, 2 open-label phase 1 clinical trials	148 melanoma patients	Autoimmune side effects associated with nivolumab treatment
4	Kroon, M.W., *et al.*	2011	Cohort	434 patients with NSV	Age of onset vitiligo, extent, duration, location, gender, family history of AI pathology, associated autoimmune diseases, TSH, fT4, ATPO
5	Pradhan, V., *et al.*	2013	Single-centre prospective observational	79 vitiligo patients	Anti-melanocyte, anti- keratinocyte, anti-thyroid ac. Antibody profile comparison India vs. Western Europe
6	Ingordo, V., *et al.*	2014	Multicentre observational	175 vitiligo patients	Serology: thyroid function, anti-thyroid Ab, thyroid ultrasound
7	Skov, J., *et al.*	2020	Cohort	110 814 twins	Co-aggregation on Hashimoto's thyroiditis, celiac disease, atrophic gastritis, Graves' disease, vitiligo, DM type 1, Addison's disease
8	van Geel, N., *et al.*	2014	Single-centre retrospective observational	700 GV-108 patients with associated	Association of AI and inflammatory pathologies in patients with GV. Phenotypic features. Complete clinical
9	Speeckaert, R.; van Geel, N.	2014	Retrospective overview	700 vitiligo patients	Distribution of forms of NSV according to clinical features
10	Ezzedine, K., *et al.*	2012	Prospective observational	679 patients: 422 post-pubertal vitiligo onset, 257 pre- pubertal	Peculiarities of NSVdistribution in the pre- and post-pubertal period. European Vitiligo Task Force Questionnaire, anti- thyroid Ab dosing, thyroid function
11	Ferrari, S.M., *et al.*	2017	Case-control	2 vitiligo patient groups and 2 controls	CXCL10 and CCL in 4 groups of patients: 50 NSV, control group without thyroiditis, 40 NSV and thyroiditis, control without NSV but with thyroiditis. Thyroid function, ultrasound, anti-thyroid Ab
12	Curti, B., *et al.*	2017	Retrospective observational	1525 cancer patients	The occurrence of irAEs after checkpoint inhibit or therapy and IL-2- prognosis
13	Ranawaka, R.R.	2014	Multicenter retrospective observational	290 patients- alopecia areata	Association of alopecia areata with otherAIpathologies
14	Olejek, A., *et al.*	2017	Prospective controlled interventional	100 patients with LS	Dynamics of associated AI pathologies after photodynamic surgery for 2 years
15	Liu, Y.H., *et al.*	2014	Case-control	481 patients with Graves' disease, control 196 people	Relationship between MIF gene polymorphism and prognosis of Graves' disease in progression to a severe form. Genotyping of all patients

Table S1. Characteristics of included studies. ATPO- anti-thyroperoxidase antibodies, fT4- free thyroxine (Free T4), TSH- thyroid stimulating hormone, NSV- non-segmental vitiligo, AI- autoimmune, Ab- antibody, DM- diabetes mellitus, GV- generalized vitiligo, CXCL10- chemokine prototype of Th1 immune response, CCL- chemokine prototype of Th2 immune response, irAEs- adverse autoimmune reactions, IL-2- interleukin 2, LS- lichen sclerosus, MIF- macrophage inhibitory factor
