# Supplementary material for: Vitiligo—Thyroid Disease Association: When, in Whom, and Why Should It Be Suspected? A Systematic Review
Source: J Pers Med. 2022 Dec 12;12(12):2048. doi: 10.3390/jpm12122048 (PMC9785784; doi:10.3390/jpm12122048)
Supplement: Supplementary file 1 [file jpm-12-02048-s001.zip › Table S2.pdf]

Table S2. Results from analyzed studies

No. Std.	Associations obtained
1	↑ level of ATPO in 43.7% of patients with stable vitiligo, 37.5% in those with progressive form
2	Pediatric onset group: family history of thyroid pathology (p=0.014) and increased duration of vitiligo (p=0.001). Adult onset group: sex F (p=0.025)
3	Vitiligo - in 8 patients with resectable melanoma (24.2%) and in 11 patients with metastatic melanoma (9.6%) Hypothyroidism in 16 patients (10.8%)
4	Thyroid function of vitiligo patients: 70 patients screened previously - 43 with thyroid dysfunction. 364 patients screened in the study - 13 patients with thyroid dysfunction, 49 with ATPO+ (>100k/uL). F>M with hypothyroidism (p<0.05). Prevalence of hypothyroidism NSV>general population (p<0.001).
5	Anti-thyrosinase (11%), ATPO (22%), anti-Tg (18%), anti-keratinocyte (24%). 3% of patients also diagnosed with other AI pathology, consistent with other studies in Indian populations vs. higher prevalence in Western European population (30%)
6	Autoimmune thyroiditis- 57 patients, of which 15 euthyroid (26.3%), 2 with clinically manifest hyperthyroidism (3.5%), 3 with clinically manifest hypothyroidism (5.3%), 3 with hyperthyroidism under therapy (5.3%), 27 with hypothyroidism under therapy (47.4%), 3 with subclinical hypothyroidism (5.3%), 2 with subclinical hyperthyroidism (3.5%)
7	To study the influence of the genetic component - study on monozygotic vs dizygotic twin pairs. AI disease co-aggregation may ↑ in monozygotic twins
8	Significantly higher percentage of body surface area affected by vitiligo in the context of associated AI thyroiditis. AI thyroiditis more common among F (p=0.005)
9	Patients with associated AI diseases, especially in those with AI thyroidit is :depigmented lesions on the wrists (p=0.001). Acral involvement more common in patients with AI thyroiditis (65/67 patients, i.e. 97% vs. 463/626, i.e. 74%)
10	Post-pubertal: only universal vitiligo, associated thyroid pathology and anti-thyroid Ac (p<0.003), stress as trigger (p<0.0001), duration of disease (p<0.0001), history of AI thyroid pathology (p=0.003), acro-facial distribution (p=0.002). Pre-pubertal: truncal (p=0.02) and limb lesions (p<0.0001).
11	CXCL10 level more ↑: NSV+ AI thyroiditis vs no AI thyroiditis (p=0.001), NSV vs NSV+ AI thyroiditis and no AI thyroiditis (p=0.001)
12	Of the 1535 cancer patients, 623 had metastatic melanoma, 919 had metastatic renal cancer and 7 had both cancers in metastatic form. 152 irAEs in 130 of the patients, is associated with a better cancer prognosis. Better tumour control in patients developing irAEs (76% vs 56%, p=0.0008). Higher survival rate if irAEs were also associated (p<0.0001). Most common irAEs- vitiligo and AI thyroiditis (70%).
13	Vitiligo in 6 patients with alopecia areata (2%), AI thyroiditis in 5 patients (1.7%). Age of onset of alopecia areata is similar in the group of patients with other associated AI pathologies vs those without associated AI pathologies. 11 out of 12 patients with associated AI pathologies have a moderate form of alopecia areata
14	The most common associated AI pathologies are AI thyroiditis (51%) and vitiligo (28%). Both women with LS who had AI pathologies prior to their LS diagnosis and those whose AI pathologies started afterwards were associated with improved
15	Coexistence of vitiligo was associated with moderate-severe Graves' disease (p=0.002). C/C and C/G alleles within the MIF gene polymorphism were associated with vitiligo in patients with untreated Graves' disease (p=0.03).

Table S2: Results from analyzed studies. ATPO- anti-thyreoperoxidase antibodies, F- female,M- male, NSV- non-segmental vitiligo, anti-Tg- anti-thyroglobulin antibodies, AI- autoimmune, irAEs- autoimmune adverse reactions, LS- lichen sclerosus, ANA- antinuclear antibodies, MIF- macrophage inhibitory factor
